# Nachhaltiges Wirtschaften setzt eine nachhaltige Arbeitswelt voraus

**DOI:** 10.1365/s35764-021-00345-8

**Published:** 2021-06-29

**Authors:** Stephan Lessenich, Marion A. Weissenberger-Eibl, Thomas Barth, Meike Walli-Schiek

**Affiliations:** 1 Institut für Soziologie, Ludwig-Maximilians-Universität München, München, Deutschland; 2 Fraunhofer-Institut für System- und Innovationsforschung ISI, Karlsruhe, Deutschland; 3 Lehrstuhl Innovations- und Technologie-Management, Karlsruher Institut für Technologie (KIT), Karlsruhe, Deutschland

Während nachhaltiger Konsum und nachhaltige Produktion längst in der Diskussion um eine zukunftsfähige Gestaltung der Wirtschaft etabliert sind, ist das Feld der Arbeit ein zwar zentraler, aber noch unterbelichteter Aspekt. Um endlich auf den Pfad einer nachhaltigen Entwicklung zu gelangen, führt kein Weg daran vorbei, auch unsere Arbeitswelt nachhaltig zu gestalten. Erst wenn nachhaltige Arbeit in den Fokus rückt, sind die Voraussetzungen geschaffen, um die Wirtschaft wirklich zukunftsfähig zu gestalten.

Nicht erst die durch Covid-19 offengelegten nationalen wie globalen Ungleichheiten und Verwundbarkeiten lassen die Nicht-Nachhaltigkeit als zutreffende Beschreibung unseres gegenwärtigen Wirtschaftens erscheinen. Schon ein Blick auf die im Jahr 2015 von den Vereinten Nationen beschlossenen Sustainable Development Goals (SDGs)^1^ und die Deutsche Nachhaltigkeitsstrategie zeigt, dass es noch ein langer Weg ist, bis auch reiche Nationen wie die Bundesrepublik Deutschland den Pfad der nachhaltigen Entwicklung einschlagen. Zwischen den Zielen und den bisherigen Fortschritten dorthin klafft noch eine gewaltige Lücke.

Nachhaltige Entwicklung ist dabei als notwendiger Teil der kontinuierlichen Veränderung von Rahmenbedingungen für Unternehmen zu begreifen – sie ist nicht verhandelbar. Die Ausrichtung an Nachhaltigkeitszielen ist also weder als bloßes Marketinginstrument noch lediglich als Belastung von Unternehmen zu verstehen. Vielmehr gilt es, der sich gegenwärtig stellenden Herausforderung, eine Nachhaltigkeitstransformation zu gestalten, mit Mut, Offenheit und Veränderungsbereitschaft zu begegnen. Nur dann können Unternehmen auch auf den Märkten der Zukunft bestehen. Die hier liegenden Chancen zu ergreifen, sollte im ureigenen Interesse von Unternehmen liegen. Es bedarf allerdings auch einer klaren politischen Gestaltung von Rahmenbedingungen, damit Unternehmen ihre Verantwortung wahrnehmen.

Nachhaltig zu produzieren und zu konsumieren, ist schon länger Teil des Zielkatalogs, um die Wirtschaft zukunftsfähig zu gestalten. Jedoch wurde erst in der jüngsten Zeit die entscheidende Schnittstelle zwischen beiden Sphären – die Arbeitswelt – ausdrücklich als Ziel 8 der SDGs in die Nachhaltigkeitsagenda aufgenommen. Die hieran ansetzenden Überlegungen gehen einerseits davon aus, dass wir die Gestaltung der Arbeitswelt nutzen müssen, um eine nachhaltige Entwicklung insgesamt voranzutreiben. Auch können so bestehende Nachhaltigkeitsblockaden und immer deutlicher hervortretende Zielkonflikte zwischen sozialen und ökologischen Nachhaltigkeitsdimensionen angegangen werden. Die Beispiele des Kohleausstiegs oder der Transformation des Mobilitätsektors belegen beispielsweise, dass die soziale Akzeptanz für die notwendigen Transformationsprozesse stark von der Einbindung in das Erwerbsarbeitssystem beeinflusst wird. Neue „grüne“ und qualitativ hochwertige Arbeitsplätze für Sektoren im Strukturwandel zu schaffen, ist dabei ein wichtiger, aber eben nur ein Baustein. Andererseits sind die Risiken hoch, dass derzeitige Veränderungen der Arbeitswelt, die mit Megatrends wie dem demografischen Wandel, der Digitalisierung und weiterer Globalisierung verbunden sind, zu weiteren nicht nachhaltigen Entwicklungen führen. Damit würden wir die Potenziale einer Transformation in Richtung nachhaltig wirtschaftender Unternehmen verschenken. Der Gestaltungsdruck ist also hoch.

Einer Agenda der nachhaltigen Arbeit zu folgen, verdeutlicht: Nachhaltig wirtschaftende Unternehmen sind jene, die entlang ihres gesamten Liefernetzwerks nachhaltige Arbeitswelten gestalten und damit eine auf guter und menschenwürdiger Arbeit basierende nachhaltige Entwicklung im Einklang mit den ökologischen Nachhaltigkeitszielen vorantreiben. Auch für die Zukunft der Arbeit gilt, ökonomische, ökologische und soziale Ziele in Balance zu bringen. Eine derartige Transformation in Richtung nachhaltiger Arbeit ist komplex und tiefgreifend, denn sie stellt die klassischen industriegesellschaftlichen Vorstellungen von Arbeit, Wirtschaft und ihre politischen Rahmenbedingungen infrage. Diese stehen jedoch ohnehin bereits unter massivem Veränderungsdruck. Die umfassende Einbeziehung bis dato externalisierter sozialer und ökologischer Kosten und die Berücksichtigung und Anerkennung auch nicht erwerbsförmiger Arbeitsformen wie Haus- und Sorgearbeit sowie ehrenamtlicher Tätigkeiten sind zentrale Bestandteile einer zukunftsorientierten, nachhaltigen Agenda. Im Folgenden skizzieren wir exemplarisch drei konkrete, auf Unternehmen bezogene Ansatzpunkte für eine solche Transformation.

## Alternative Arbeitsformen in nachhaltig wirtschaftenden Unternehmen erproben

Nicht bezahlte Tätigkeiten, etwa Hausarbeit oder die Sorge für und Pflege von Angehörigen, aufzuwerten und nicht gewinnorientierte Unternehmensformen zu fördern, spielt eine wichtige Rolle in der Nachhaltigkeitsdebatte. Zudem sollten in bestehenden Unternehmen alternative Arbeitsformen ausprobiert werden, um nachhaltiges Wirtschaften zu fördern. So sollten beispielsweise Unternehmen unter wissenschaftlicher Begleitung die Vor- und Nachteile des mobilen Arbeitens gezielt erproben – und zwar sowohl unter sozialen (z. B. Vereinbarkeit von Beruf und Familie, freie Wahl des Arbeitsortes), ökologischen (z. B. weniger Verkehr, weniger Emissionen durch [Reise-]Verkehr) als auch wirtschaftlichen (z. B. statt Reisen mehr Zeit für relevante Aufgaben, effizienteren Einsatz der Kapazitäten ohne Arbeitsintensivierung) Gesichtspunkten. Die aktuelle Situation hat mit der erheblichen Ausweitung von mobilen Arbeitsformen Möglichkeitsräume eröffnet, die es zu nutzen gilt. Hierfür ist der Austausch von Wissenschaft, Belegschaften und Management notwendig – denn nur so bringen wir die unterschiedlichen Perspektiven und Bedarfe zusammen und können gemeinsam kreative und nachhaltige Lösungen entwickeln und umsetzen.

Auch digitale Arbeit – mit und im Internet oder durch selbstarbeitende Technik, innerhalb und außerhalb fester betrieblicher Strukturen – muss im Sinne guter und nachhaltiger Arbeit gestaltet sein. Gerade auch dann, wenn sie innerhalb des Liefernetzwerks nicht in der Form klassischer, sozialpolitisch abgesicherter Arbeitsverhältnisse stattfindet.

## Digitalisierung sozialökologisch nachhaltig gestalten

Eine Studie zeigt etwa am Beispiel von Mobilitätsdienstleistern und mobilen Diensten, dass Apps und Algorithmen mittlerweile nicht nur Arbeitsaufträge erteilen, sie kontrollieren zudem. Hierdurch entsteht eine hohe Abhängigkeit der Arbeitenden (tendenziell als Solo- und Scheinselbstständige) von technischen Systemen. Auch verlagern sich Verantwortung und Risiken tendenziell auf die Beschäftigten und Gestaltungsspielräume werden eingeschränkt. Einer Fremdbestimmung durch Algorithmen beispielsweise können Erwartungen an Selbstverwirklichung und Sinnstiftung gegenüberstehen. Gleichzeitig entwickeln sich durch die Digitalisierung Berufsbilder, die Beschäftigten Spielräume für Kreativität und Entscheidungen geben. Daneben können intelligente Maschinen Menschen weltweit entlasten, die unter extremen und gefährlichen Bedingungen arbeiten. Für Beschäftigte mit geringer Qualifikation jedoch erhöht sich das Risiko, durch Automatisierung den Arbeitsplatz zu verlieren. Daher ist es essenziell, auch die nicht nachhaltigen Entwicklungen der Arbeit in den Blick zu nehmen und zu diskutieren. Sonst laufen wir Gefahr, die Potenziale für zukunftsfähiges und erfolgreiches Wirtschaften zu verschenken.

## Beschäftigte für einen Wandel hin zu nachhaltig wirtschaftenden Unternehmen einbinden

Beschäftigte sind Expert*innen, wenn es darum geht, Wandel in Unternehmen voranzutreiben – auch für eine Transformation hin zu nachhaltigen Unternehmensprozessen und -strukturen. Es gilt daher, die Beschäftigten eng einzubinden, unter anderem indem sie sich ideengebend und mitgestaltend einbringen können und ihre Vorschläge Gehör finden. Denn Innovation steckt im Menschen und zwar unabhängig von der Ausbildung, der Tätigkeit oder der Stellung im Unternehmen. So schafft der interdisziplinäre Austausch die Möglichkeit, Ideen für nachhaltige Prozesse zu generieren, gewohnte Pfade zu verlassen und nachhaltige Wege zu gehen. Gemeinsames, auf Nachhaltigkeitsprobleme zielendes Denken braucht aber Gelegenheiten. Diese können und müssen Unternehmen im Austausch mit den Beschäftigten und ihren Interessenvertretungen schaffen. Das „Ideenmanagement“ beispielsweise bietet Unternehmen verschiedene Ansätze und Methoden für die Generierung, Sammlung und Auswahl von Ideen für mehr Nachhaltigkeit im Unternehmen.

Entscheidend ist hier eine Unternehmenskultur, die offen ist für neue Ideen und Wandel und die Experimentierräume zur Erreichung verschiedener Nachhaltigkeitsziele bietet (u. a. Geschlechtergleichheit, lebenslanges Lernen, menschenwürdige Arbeit, Innovation). Die Voraussetzung hierfür besteht in der Stärkung demokratischer Strukturen der Mitgestaltung in Unternehmen, die sowohl die Mitbestimmung von Beschäftigten erleichtert und unterstützt als auch den Austausch mit der Gesellschaft und den Bürger*innen ermöglicht. Denn auch in der Zukunft einer Nachhaltigkeitstransformation sollten sich erfolgreiche Unternehmen als Teil von sozialen Gemeinschaften begreifen, die ihren Fortbestand sichern.

## Wege zu einer nachhaltigen Arbeitswelt

In Sachen Arbeitswelt stehen wir vor gewaltigen Gestaltungsaufgaben, damit eine nachhaltige Entwicklung gelingt – und die Corona-Krise führt uns einmal mehr die Dringlichkeit vor Augen. Wichtig wird dabei insbesondere sein, die Arbeitswelt nicht nur punktuell zu reformieren, sondern umfassend und nachhaltig zu transformieren, sodass das Soziale, Wirtschaftliche und Ökologische zukünftig wie selbstverständlich zusammen gedacht und gestaltet werden kann. In ihrem Papier „*Wege zu einer nachhaltigen Arbeitswelt*“ beleuchtet die Arbeitsgruppe „Zukunft der Arbeit“ der „Wissenschaftsplattform Nachhaltigkeit 2030“ über die oben genannten Aspekte hinaus unter anderem den Status von Arbeit und langfristige Trends in der Arbeitswelt. Neben einem umfassenden Konzept für nachhaltige Arbeit bedarf es dringend struktureller Veränderungen. Da derartige tiefgreifende Veränderungsprozesse stets von Interessenkonflikten gekennzeichnet sind, müssen wir in Deutschland den gesellschaftlichen Dialog zu Arbeit und Nachhaltigkeit intensiver führen. Daneben präsentiert die Arbeitsgruppe erste Vorschläge für Gütekriterien nachhaltiger Arbeit, die in einem gesamtgesellschaftlichen Dialog aufgegriffen und weiter konkretisiert werden sollten.

### Zusammenfassung

- Nicht-Nachhaltigkeit ist noch immer ein Strukturmerkmal gegenwärtigen Wirtschaftens. Die bisher unterbelichtete Voraussetzung für ein Umsteuern ist die Schaffung einer nachhaltigen Arbeitswelt.
- Für eine nachhaltige Arbeitswelt müssen viele ihrer Bereiche grundsätzlich transformiert werden.
- Die Gestaltung einer nachhaltigen Arbeitswelt kann auch übergeordnet dazu beitragen, Zielkonflikte zwischen sozialen und ökologischen Nachhaltigkeitsdimensionen zu lösen.

### Kernthesen

- Nachhaltige Entwicklung ist als notwendiger Teil der kontinuierlichen Veränderung von Rahmenbedingungen für Unternehmen zu begreifen – sie ist nicht verhandelbar.
- Nachhaltig wirtschaftende Unternehmen sind jene, die entlang ihres gesamten Liefernetzwerks nachhaltige Arbeitswelten gestalten und damit eine auf guter und menschenwürdiger Arbeit basierende nachhaltige Entwicklung im Einklang mit den ökologischen Nachhaltigkeitszielen vorantreiben.
- Transformation in Richtung nachhaltiger Arbeit ist komplex und tiefgreifend, sie stellt die klassischen industriegesellschaftlichen Vorstellungen von Arbeit, Wirtschaft und ihre politischen Rahmenbedingungen infrage.

### Handlungsempfehlungen

- Bislang unterbelichtete nachhaltigkeitsrelevante Dimensionen von Arbeit stärker in den Fokus rücken, etwa nicht bezahlte Arbeitsformen wie Hausarbeit, Sorgearbeit und ehrenamtliche Tätigkeiten
- Nachhaltige Gestaltung der Digitalisierung: Chancen nutzen, Risiken ernst nehmen
- Einbeziehung der Beschäftigten in Transformationsprozesse – auch durch demokratische Beteiligungsformen

## Footnotes

1 Siehe auch: https://17ziele.de/

